# Monitoring Src status after dasatinib treatment in HER2+ breast cancer with ^89^Zr-trastuzumab PET imaging

**DOI:** 10.1186/s13058-018-1055-2

**Published:** 2018-10-25

**Authors:** Brooke N. McKnight, Nerissa T. Viola-Villegas

**Affiliations:** Department of Oncology, Karmanos Cancer Institute, 4100 John R Street, Detroit, MI 48201 USA

**Keywords:** Src, pSrc, pHER2, HER2, Dasatinib, PET, Trastuzumab, Breast cancer

## Abstract

**Background:**

De novo or acquired resistance in breast cancer leads to treatment failures and disease progression. In human epidermal growth factor receptor 2 (HER2)-positive (HER2+) breast cancer, Src, a non-receptor tyrosine kinase, is identified as a major mechanism of trastuzumab resistance, with its activation stabilizing aberrant HER2 signaling, thus making it an attractive target for inhibition. Here, we explored the causal relationship between Src and HER2 by examining the potential of ^89^Zr-trastuzumab as a surrogate imaging marker of Src activity upon inhibition with dasatinib in HER2+ breast cancer.

**Methods:**

HER2+ primary breast cancer cell lines BT-474 and trastuzumab-resistant JIMT-1 were treated with dasatinib and assessed for expression and localization of HER2, Src, and phosphorylated Src (pSrc) (Y416) through western blots and binding assays. Mice bearing BT-474 or JIMT-1 tumors were treated for 7 or 14 days with dasatinib. At the end of each treatment, tumors were imaged with ^89^Zr-trastuzumab. The results of ^89^Zr-trastuzumab positron emission tomography (PET) was compared against tumor uptake of fluorodeoxyglucose (^18^F-FDG) obtained the day before in the same group of mice. Ex vivo western blots and immunohistochemical staining (IHC) were performed for validation.

**Results:**

In BT-474 and JIMT-1 cells, treatment with dasatinib resulted in a decrease in internalized ^89^Zr-trastuzumab. Confirmation with immunoblots displayed abrogation of pSrc (Y416) signaling; binding assays in both cell lines demonstrated a decrease in cell surface and internalized HER2-bound tracer. In xenograft models, dasatinib treatment for 7 days (BT-474, 11.05 ± 2.10 % injected dose per gram of tissue %(ID)/g; JIMT-1, 3.88 ± 1.47 %ID/g)) or 14 days (BT-474, 9.20 ± 1.85 %ID/g; JIMT-1, 4.45 ± 1.23 %ID/g) resulted in a significant decrease in ^89^Zr-trastuzumab uptake on PET compared to untreated control (BT-474, 17.88 ± 2.18 %ID/g; JIMT-1, 8.04 ± 1.47 %ID/g). No difference in ^18^F-FDG uptake was observed between control and treated cohorts. A parallel decrease in membranous HER2 and pSrc (Y416) staining was observed in tumors post treatment on IHC. Immunoblots further validated the ^89^Zr-trastuzumab-PET readout. Positive correlation was established between ^89^Zr-trastuzumab tumor uptake versus tumor regression, pSrc and pHER2 expression.

**Conclusions:**

^89^Zr-trastuzumab can potentially assess tumor response to dasatinib in HER2+ breast cancer and could be used as a surrogate tool to monitor early changes in Src signaling downstream of HER2.

**Electronic supplementary material:**

The online version of this article (10.1186/s13058-018-1055-2) contains supplementary material, which is available to authorized users.

## Background

The human epidermal growth factor receptor 2 (HER2) has become a critical therapeutic target with trastuzumab as the mainstream, first-in-line standard of care in patients with HER2-positive breast cancer [1, 2]. Unfortunately, response rates to HER2-targeted therapy remain dismal due to acquired and de novo resistance, which in part can be attributed to alterations in receptor tyrosine kinases (RTKs) [3], and downstream signaling transduction pathways, such as Src [4, 5].

Src is a non-receptor tyrosine kinase expressed ubiquitously that interacts with several RTKs [6]. Its activation enhances cellular migration and survival [7]. Elevated Src has been shown to stabilize HER2 and vice versa [6, 8–10], establishing a functional relationship between the two oncogenes [8]. This was reported in a study by Fan et al. wherein Src abrogation concomitantly led to decreased HER2 levels within 7–14 days of treatment with a Src inhibitor, PP2, in vitro [9]. Thus, Src is implicated as one of the key molecules driving resistance to trastuzumab therapy, making this signaling axis an attractive target for inhibition.

Dasatinib (Sprycel®) is a Src and BCR/ABL tyrosine kinase inhibitor, which was approved by the Food and Drug Administration (FDA) for treatment of leukemia in 2006 [11]. Preclinical data reported by Seoane et al. demonstrated the synergistic effects of dasatinib with trastuzumab as evidenced by attenuated phosphorylated levels of Src, extracellular signal-related protein kinase (ERK) and protein kinase B (Akt) in HER2+ breast cancer [12]. These preclinical findings were validated in a prospective phase I–II trial exploring the combined efficacy and safety of dasatinib, trastuzumab and paclitaxel in patients with breast cancer [13]. Monitoring of tumor response to this drug cocktail was conducted through immunohistochemical analysis (IHC) of patients’ skin samples. However, better ways to non-invasively monitor tumor response can be achieved by exploring the direct causal relationship between HER2 and Src.

In this study, we investigated the potential of ^89^Zr (half-life (t_1/2_) ~ 3.27 days) labeled trastuzumab (Herceptin®) as a surrogate tool to monitor biologic effects of dasatinib treatment in HER2-positive (HER2+) breast cancer. We first evaluated the specificity of ^89^Zr-trastuzumab in both HER2+ and Src-active breast cancer cell lines, BT-474 and JIMT-1, which are trastuzumab-sensitive and trastuzumab-resistant, respectively. MDA-MB-468 triple-negative breast cancer cell line was used as a control. We next examined the utility of fluorodeoxyglucose (^18^F-FDG) and ^89^Zr-trastuzumab as a predictive imaging tool using the same group of mice bearing either BT-474 or JIMT-1 tumors treated with dasatinib. After imaging, a correlation was tested between ^89^Zr-trastuzumab positron emission tomography (PET) uptake and changes in tumor volume, immunoblots, and IHC analysis.

## Methods

### Cell lines, reagents, and xenografts

BT-474 and JIMT-1 cells were a generous gift from Prof. Jason S. Lewis at Memorial Sloan Kettering Cancer Center (MSKCC). MDA-MB-468 cells were provided by the Karmanos Cancer Institute (KCI) Biobanking and Correlative Sciences (BCS) Core. BT-474 cells were grown in 1:1 DMEM:F12 (VWR) + 5% FBS + 1% Pen-Strep + 1% non-essential amino acids (NEAA) (Corning); JIMT-1 and MDA-MB-468 cells were grown in DMEM + 1% Pen-strep + 5% FBS (Sigma). All cells were grown at 37 °C with 5% CO_2_ and routinely tested for mycoplasma with MycoAlert Mycoplasma Detection Kit (Lonza). The cell lines were further authenticated by the Biobanking and Correlative Services Core at Wayne State University. Dasatinib (Selleckchem) was prepared as a 50-mM stock concentration in dimethyl sulfoxide (DMSO) and serially diluted to the desired concentration with cell culture medium for in vitro assays. Trastuzumab was obtained from the Karmanos Cancer Center pharmacy. Primary antibodies for western blots and IHC are commercially available and are detailed in Additional file 1: Table S1. Anti-rabbit and anti-mouse HRP-linked secondary antibodies were purchased from GE (NA934, NA931).

All animal handling and manipulations were conducted in accordance with the guidelines set by Wayne State University Institutional Animal Use and Care Committee. For imaging experiments, female athymic nu/nu mice (6–8 weeks old, Envigo) were subcutaneously (s.c.) injected with 5 × 10^6^ MDA-MB-468 or 5 × 10^6^ JIMT-1 breast cancer cells. For BT-474 xenografts, mice were implanted s.c. with 0.72 mg slow-release 60-day 17-β Estradiol pellets (SE-121, Innovative Research of America) on the nape of the neck for 2–3 days before 10 × 10^6^ cells were injected. All cells were injected as a suspension in 150 μL 1:1 medium:Matrigel® (BD Biosciences, Bedford, MA, USA) on the right shoulder. Monitoring of tumor growth was performed weekly with calipers. The tumor volume was calculated using the formula: length × width × height × π/6. Mice with tumor volumes ranging from 150 to 250 mm^3^ were utilized.

### Radiosynthesis of ^89^Zr-trastuzumab

p-Benzyl-isothiocyanate-desferrioxamine (DFO, Macrocylics, Inc.) was conjugated to trastuzumab and a non-specific human IgG isotype (14506, Sigma-Aldrich) according to published protocols [14, 15]. The synthesis was performed using 4:1 mol equivalence of DFO-Bz-SCN to trastuzumab or IgG, respectively in 0.9% saline, pH ~ 9 at 37 °C for 1 h. Pure, monoclonal antibody DFO-conjugates were obtained by passing through a spin column filter with a molecular weight cutoff of 30 kDa (GE Vivaspin 500) using sterile saline as eluting buffer.

Approximately 1 mCi (37 MBq) of ^89^Zr-oxalate (3D Imaging, LLC) was neutralized to pH 7.0–7.2 using 1 M NaOH. Trastuzumab-DFO (200 μg) was added to the ^89^Zr solution. The reaction was quenched after 1–1.5 h incubation at room temperature upon addition of 5 μL of 50 mM EDTA (pH ~ 7) to eliminate any non-specifically bound ^89^Zr. Radiolabeling efficiency > 95% was determined by radio-instant thin layer chromatography (iTLC) using silica gel-impregnated iTLC strip (Agilent Technologies, Santa Clara, CA, USA) and 50 mM EDTA as the solid and mobile phase, respectively. Pure ^89^Zr-trastuzumab was obtained through spin column centrifugation (GE Vivaspin 500, MWCO: 30 kDa) with saline used for eluting unbound radiometal. Radiochemical purity > 99% was achieved based on iTLC analysis. ^89^Zr-trastuzumab was assessed for immunoreactivity as previously described [16]. Radiolabeling and purification of a non-specific human IgG monoclonal antibody was performed as aforementioned.

### IC_50_ Calculations

Half-maximal inhibitory concentration (IC_50_) values were obtained for BT-474 and JIMT-1 breast cancer cell lines. Wells were seeded with ~ 1 × 10^4^ cells and incubated overnight at 37 °C in 5% CO_2_. Cells were treated with increasing concentrations of dasatinib (1 nM to 1 mM) and incubated for 72 h then analyzed for viability using alamar blue assay (Life Technologies). After 4 h incubation with alamar blue, absorbance was read at 570 nm on an Infinite M200 plate reader (Tecan). IC_50_ was calculated in GraphPad Prism (v. 7.02) using a non-linear dose response plotting the  log(concentration) versus % viable cells.

### Internalization assay

Wells were seeded with 50,000 cells and incubated overnight. Cells were treated with the established IC_50_ for dasatinib in complete medium for 0–48 h. After incubation, radiolabeled protein (100 ng, 0.30 μCi, 111 kBq) in 1 mL of medium was added to each well. The plates were incubated at 37 °C for 2 h. Following the incubation period, the medium was collected and the cells were rinsed with 1× PBS twice. Surface-bound activity was removed by washing the cells in 100 mM acetic acid + 100 mM glycine (1:1, pH 3.5) at 4 °C. The cells were then lysed with 1 M NaOH. All washes (medium plus PBS, acid and alkaline) were collected in separate tubes and measured for bound activity using a gamma counter (Perkin Elmer). The percentage of internalized activity was calculated as the ratio of the activity of the lysate and the total activity collected from the medium plus PBS, and base washes, normalized to 50,000 cells counted using a Countess II Automated Cell Counter (Thermo Fisher).

### In vitro competitive binding assay

Binding of ^89^Zr-trastuzumab was evaluated in all three cell lines. Wells were seeded with 10,000 cells and incubated overnight. After incubation, radiolabeled protein (1 μCi/mL, 37 kBq/mL, 0.25 μg) in 1 mL of medium was added to each well with or without 10-fold excess unlabeled trastuzumab (1 μg). The plates were incubated at 4 °C for 1 h. Following the incubation period, the medium was collected and the cells were rinsed with 1 mL 1 × PBS twice. The cells were then lysed with 1 mL 1 M NaOH. All washes (medium including PBS and alkaline wash) were collected in separate tubes and measured for counts using a gamma counter (Perkin Elmer). The percentage of bound activity was calculated as the ratio of the activity of the lysate and the total activity collected from the medium plus PBS, and base washes, and was normalized to cell count using a Countess II Automated Cell Counter (Thermo Fisher).

### Immunoblots

After dasatinib treatment, cells were lysed on ice using 1 × RIPA buffer (Pierce) supplemented with HALT protease and phosphatase inhibitor cocktail (Pierce). Tumors were mechanically lysed using a handheld homogenizer Polytron PE 1200E (VWR) in the same buffer. Total protein was measured using a Pierce BCA Protein Assay Kit (Thermo Fisher).

Proteins were separated on a 4–12% bis-tris NuPAGE gel (Invitrogen) before transferring to an Immobilon-P polyvinylidene di fluoride (PVDF) membrane (Millipore Sigma). Membranes were blocked in 5% non-fat dry milk in Tris-buffered saline and Tween 20 (TBST) buffer for 1 h at room temperature. Primary antibodies (Additional file 1: Table S1) were diluted in TBST with 0.02% sodium azide and incubated at 4 °C before blotting with HRP-linked secondary antibodies in 5% milk-TBST for 2 h at room temperature. Proteins were visualized using Amersham ECL (GE) with images collected and analyzed using a ChemiDoc (BioRad) system with Image Lab (Bio-Rad) software. Densitometry was calculated using ImageJ.

### Mouse treatment studies

Dasatinib (75 mg/kg body weight in 150 μL 1:1 sterile water:glycerol) was administered to BT-474 and JIMT-1 tumor-bearing mice by oral gavage for 7 and 14 days. Untreated control mice were given a 1:1 mix of water and glycerol (150 μL total volume via oral gavage) as placebo. Food and water were given *ad libitum*. Tumor volumes were recorded 2–3 times per week. Percent change in tumor volume was analyzed using measurements obtained before the start of treatment and at the time of imaging.

### PET imaging and distribution

On the last day of treatment, mice bearing BT-474 or JIMT-1 tumors (*n* = 3–4) were fasted 8 h before intravenously (i.v.) administering ^18^F-FDG (150–200 μCi, 5.55–7.4 MBq). The mice were anesthetized with 1–2% isoflurane immediately after tracer injections. PET scans were acquired 1 h post-injection (p.i.). After 24 h, ^89^Zr-trastuzumab (200–240 μCi, 7.40–8.88 MBq, 67–80 μg, 40–50 nmol) in sterile saline was administered i.v. in the same group of mice. Small-animal PET scans were acquired at 48 h p.i. using a microPET-R4 scanner (Concorde Microsystems). All mice were fully anesthetized with 1–2% isoflurane (Baxter, Deerfield, IL, USA) during each scan acquisition. Images were reconstructed via filter back projection. ASIPro VM™ software (Concorde Microsystems) was used to analyze volumes of interest (VOI) on various planar sections from the acquired image by manually drawing on the tumor site and on select organs. The average VOI was calculated and expressed as the percentage of injected dose per gram of tissue (%ID/g). Mice were euthanized after imaging via CO_2_ asphyxiation and cervical dislocation. Tumors were removed and immediately snap frozen in liquid nitrogen and stored at − 80 °C until decayed (~ 35 days).

^89^Zr-trastuzumab biodistribution was performed at 48 h p.i. of the tracer (20–30 μCi, 0.74–1.11 MBq, 336–504 nmol, 5–7 μg) in mice bearing BT-474 or JIMT-1 tumors. ^89^Zr-IgG (20–30 μCi, 0.74–1.11 MBq, 5-7 μg, 336–504 nmol) was injected in a separate group of tumor-bearing mice to assess non-specific accumulation of the tracer. Mice were sacrificed as stated above. Bound activity was measured on tissues of interest using a gamma counter (Perkin Elmer Wizard2) and is expressed as the percentage of injected dose per gram of tissue (wet weight).

### Immunohistochemical analysis

Tumors embedded in optimal cutting temperature (OCT) blocks were sliced into 5-μm sections (Leica CM 1850), mounted on positively charged slides (Fisher) and dried overnight at room temperature. Slides were fixed in pre-cooled acetone for 10 min and allowed to evaporate ~20 min. Endogenous activity was blocked with 0.3% H_2_O_2_ for 10 min, before incubation with 10% FBS in PBS for 1 h in a humidified chamber at room temperature. Tissues were immunostained for HER2 and pSrc (Y416) (Additional file 1: Table S1) using a Histomouse Max broad spectrum 3,3-diaminobenzidine (DAB) kit (Invitrogen). Slides were scanned using a Leica SCN 400 slide scanner with image viewer software.

### Statistical analysis

Statistical analysis was performed by two-way analysis of variance (ANOVA) using GraphPad Prism v. 7.02. A value of *p* < 0.05 was considered statistically significant. Data were expressed as the mean ± S.D.

## Results

### Characterization of ^89^Zr-trastuzumab

High radiolabeling yields (> 95%) were obtained with > 97% purity after purification via spin column. Specific activity of 3.0 ± 0.2 mCi/mg (111 ± 7.4 Bq/μmol) was established. The labeled antibody retained immunoreactivity towards HER2 with 85% retention (Additional file 2: Figure S1, *n* = 3).

### In vitro treatment studies with dasatinib

**Fig. 1:**
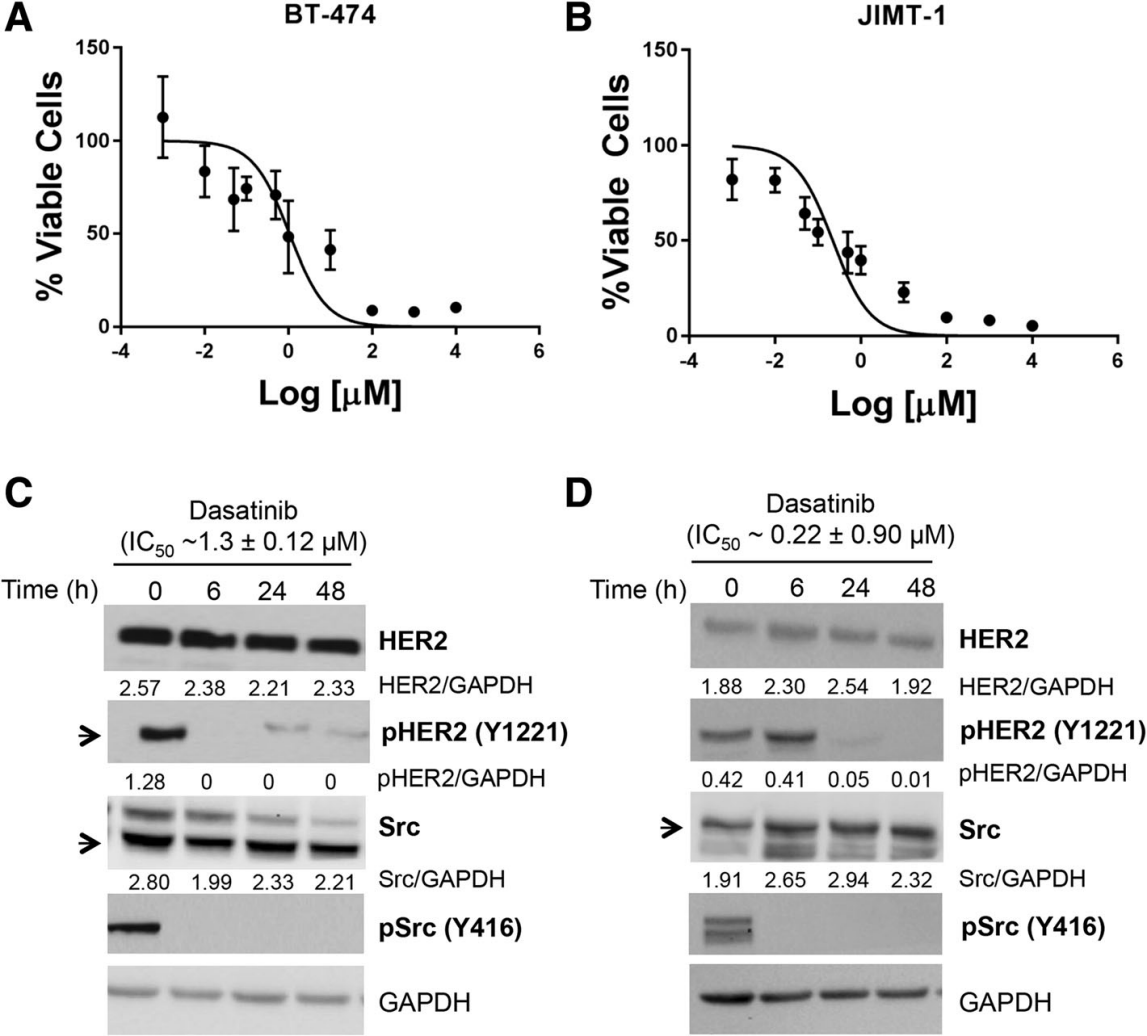
Dasatinib treatment decreases phosphorylated Src (pSrc) (Y416) and phosphorylated human epidermal growth factor receptor 2 (pHER2) (Y-1221) protein levels in vitro. BT-474 (**a**) or JIMT-1 (**b**) cells were treated with increasing concentrations of dasatinib for 72 h to achieve half maximal inhibitory concentration (IC_50_) values of 1.3 ± 0.12 μM and 0.22 ± 0.09 μM, respectively. BT-474 cells (**c**) and JIMT-1 (**d**) were treated with the IC_50_ dasatinib up to 48 h and western blots were performed for HER2, Src, pSrc (Y416), and pHER2 (Y1221/1222). Densitometry results are shown as the ratio of target protein/glyceraldehyde-3-phosphate dehydrogenase (GAPDH)

BT-474 (Fig. 1a) and JIMT-1 (Fig. 1b) cells were treated with increasing concentrations of dasatinib to achieve IC_50_ values of 1.3 ± 0.12 μM, and 0.22 ± 0.09 μM, respectively, 72 h post-treatment. Treated and control groups of cells were lysed and analyzed by western blot. In BT-474 cells (Fig. 1c), total abrogation of pSrc (Y416, directly associated with full tyrosine kinase activity) [17, 18] and pHER2 (Y1221/1222, autophosphorylation site) were observed after 6 h of exposure. While in JIMT-1 cells (Fig. 1d), attenuation of pSrc (Y416) activity after 6 h and pHER2 (Y1221/1222) at 24 h was displayed post dasatinib treatment. No changes in total HER2 or Src protein levels were observed for either cell line as shown by densitometry.

### Src treatment lowers ^89^Zr-trastuzumab internalization

**Fig. 2:**
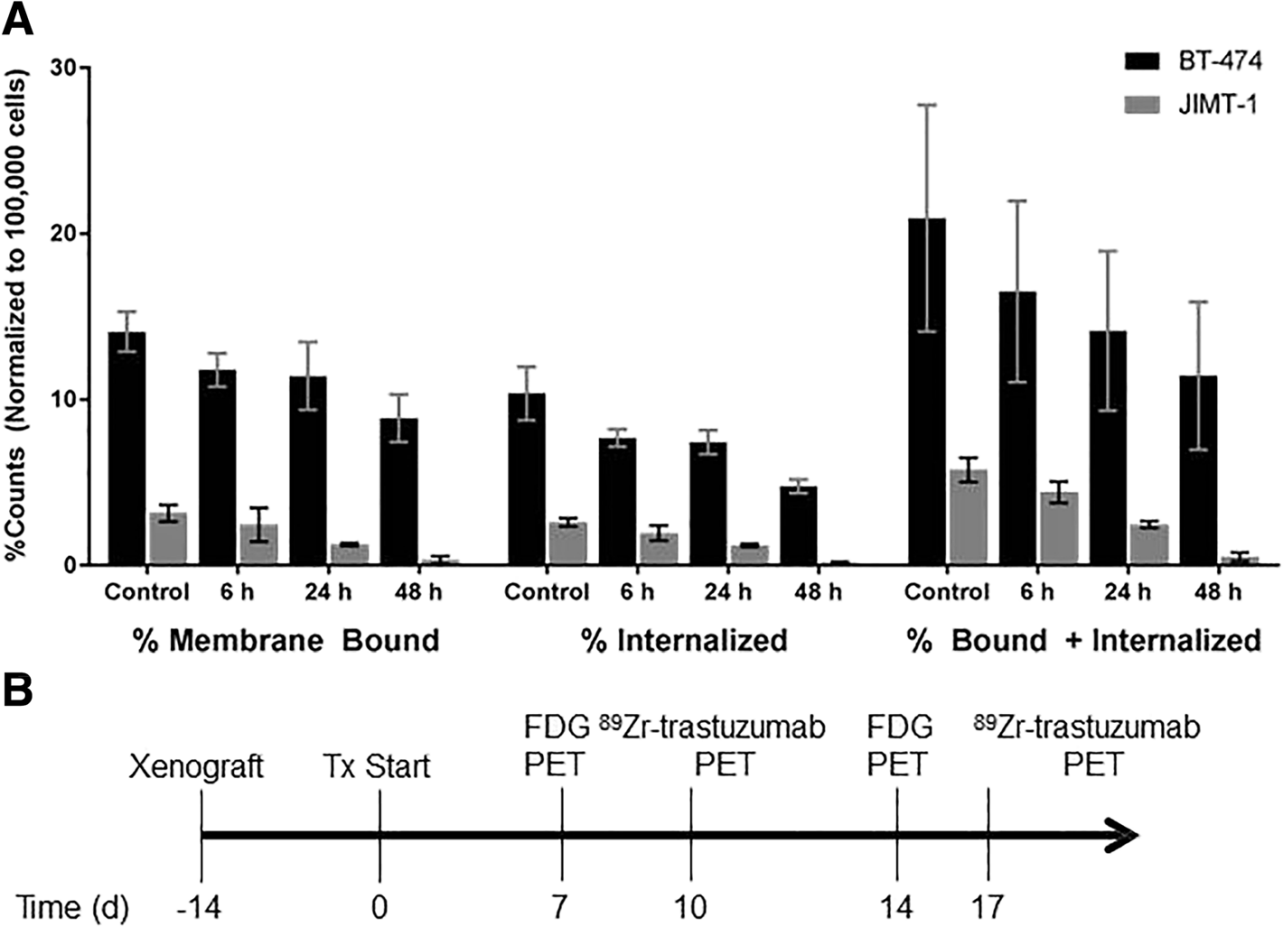
^89^Zr-trastuzumab binding and uptake decreases upon dasatinib treatment. Internalization and binding assays of ^89^Zr-trastuzumab on BT-474 and JIMT-1 cells treated with dasatinib half maximal inhibitory concentration (IC_50_) from 0 to 48 h showed a decrease in probe internalization and binding over time (**a**). Treatment and imaging scheme illustrates treatment of tumors for 7 days and/or 14 days with dasatinib followed by positron emission tomography (PET) with fluorodeoxyglucose (^18^F-FDG). ^89^Zr-trastuzumab was administered a day after with imaging acquired 48 h post injection (**b**). Tx, treatment; d, days

We next interrogated the ability of HER2 to internalize ^89^Zr-trastuzumab after dasatinib treatment over time (Fig. 2a). A steady decrease in ^89^Zr-trastuzumab internalization was exhibited by both cell lines. Internalization of ^89^Zr-trastuzumab in untreated BT-474 was measured at 10.37 ± 1.62%; however, internalized fractions decreased after 6 h and 24 h of dasatinib treatment with ~ 7.68 ± 0.53% (*p* = 0.02), and 7.42 ± 0.74% (*p* = 0.03), respectively. At 48 h, only ~ 4.78 ± 0.42% (*p* = 0.006) of the radiotracer was found intracellularly. JIMT-1 cells also showed a reduction in internalization upon treatment, albeit after extended drug exposure. From 2.6 ± 0.25% internalized in untreated cells, no significant internalized fractions were observed at 6 h (1.96 ± 0.46%, *p* = 0.10). At prolonged treatment times, a reduction in internalized activity was observed (24 h, 1.22 ± 0.10%, *p* = 0.009 and 48 h, 0.17 ± 0.5%, *p* < 0.0001).

Membrane-bound levels of ^89^Zr-trastuzumab displayed a similar trend to internalized fractions of the imaging probe during dasatinib treatment (Fig. 2a). Compared to untreated BT-474 cells with 14.10 ± 1.22% surface-bound radiotracer, a decrease was observed in treated groups after 24 h (11.42 ± 2.04%, *p* = 0.038) and 48 h (8.88 ± 1.44%, *p* = 0.0002) of treatment. Concurrent lower tracer binding was observed in JIMT-1 cells but at longer incubation with dasatinib (48 h, 0.34 ± 0.21%, *p* = 0.028) relative to untreated cells at 3.16 ± 0.50%.

These findings are in good agreement with the western blot results wherein abrogration of pHER2 (Y1221/1222) after treatment over 6 h in BT-474 and 24 h in JIMT-1 corresponded to reduced internalization of the tracer at these time points. Collectively, this study suggests an association between decreased cell-surface binding and functional internalization of ^89^Zr-trastuzumab (percentage bound and internalized) and response to dasatinib treatment.

### Validation of ^89^Zr-trastuzumab specificity to HER2

In the in vitro studies using BT-474, JIMT-1, and MDA-MB-468 cells, co-administration of 25-fold unlabeled trastuzumab exhibited lower binding of ^89^Zr-trastuzumab in HER2+ cell lines (BT-474, 1.07 ± 0.24% vs. 6.64 ± 1.14%, *p* < 0.0001; JIMT-1, 0.65 ± 0.18 vs. 1.46 ± 0.24, *p* = 0.0007). No change in probe uptake was observed in the HER2- MDA-MB-468 cells (0.71 ± 0.40 vs. 1.11 ± 0.56, *p* = 0.34) (Additional file 3: Figure S2A).

Mice bearing BT-474, JIMT-1, or MDA-MB-468 xenografts were imaged with ^89^Zr-trastuzumab at 48 h p.i. (Additional file 3: Figure S2B, C). MDA-MB-468 tumors exhibited the lowest uptake with 3.9 ± 0.6 %ID/g, compared to BT-474 (17.9 ± 2.2 %ID/g, *p* < 0.001) and JIMT-1 (7.7 ± 0.6 %ID/g, *p* < 0.001) tumors. There was significantly less ^89^Zr-trastuzumab uptake in JIMT-1 tumors compared to BT-474 (*p* < 0.0001).

The specificity of the HER2-specific tracer was further challenged using ^89^Zr-IgG through distribution studies. In BT-474 tumors, ^89^Zr-trastuzumab uptake was 16.01 ± 3.78 %ID/g, compared to the non-specific probe (1.02 ± 0.87 %ID/g, *p* = 0.0002) (Additional file 4: Figure S3, Additional file 5: Table S2). Accumulation of ^89^Zr-trastuzumab (4.13 ± 2.36 %ID/g) in JIMT-1 xenografts of (Additional file 4: Figure S3, Additional file 6: Table S3) was significantly higher than ^89^Zr-IgG with 0.79 ± 0.24 %ID/g (*p* = 0.034).

### In vivo monitoring of tumor response to dasatinib

**Fig. 3:**
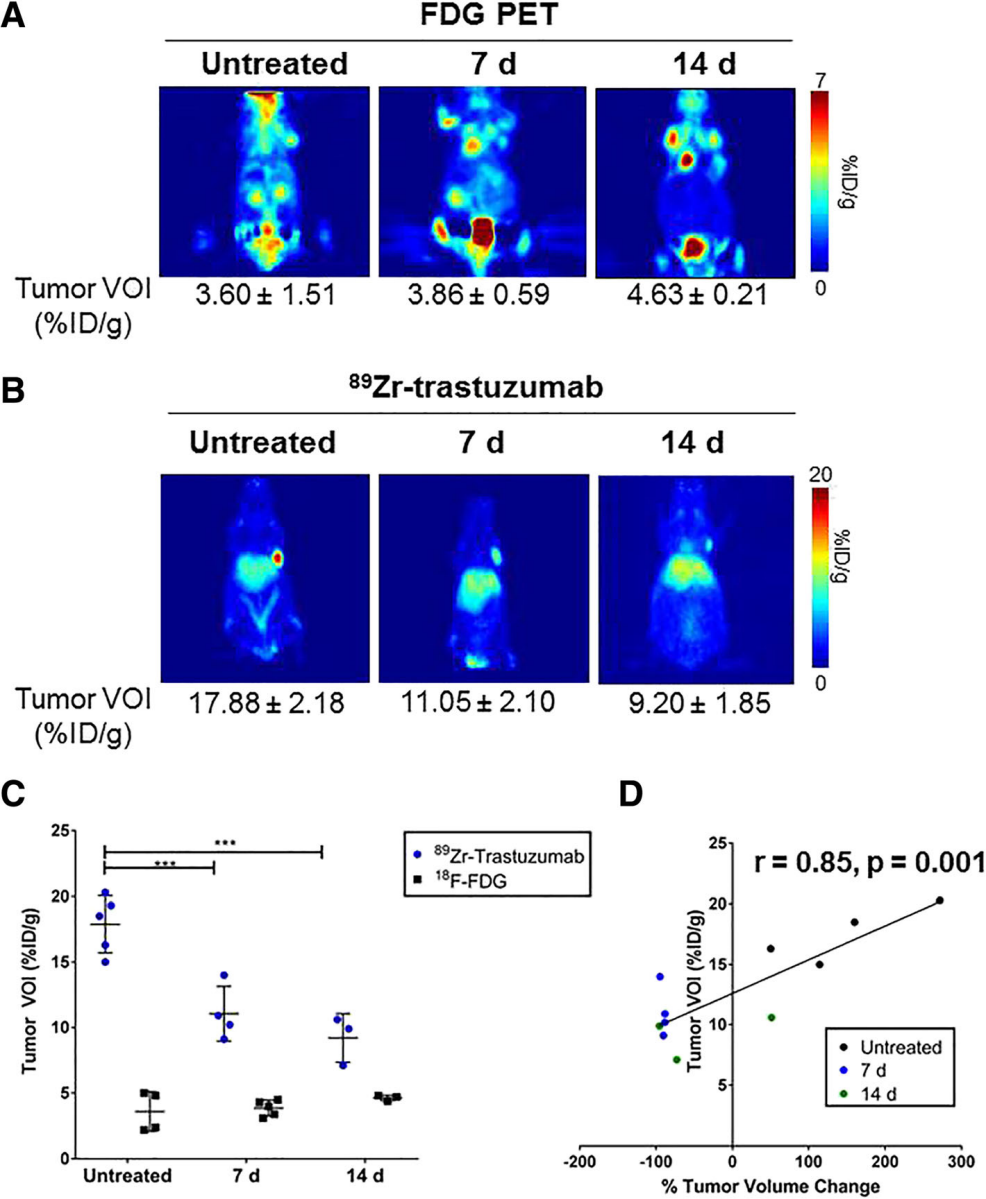
^89^Zr-trastuzumab positron emission tomography (PET) predicts tumor response to treatment in BT-474 xenografts. Untreated (left) and treated BT-474 tumors for 7-day (middle) or 14-day (right) treatment with 75 mg/kg/body weight dasatinib were imaged with fluorodeoxyglucose (FDG)-PET (**a**). In the same group of mice, PET with ^89^Zr-trastuzumab demonstrated attenuated tracer accumulation in treated groups compared to control (**b**). Tumor volumes of interest (VOIs) demonstrated lower tumor uptake of ^89^Zr-trastuzumab in treated groups compared to control; no observed changes were detected by FDG in the control or treated groups (**c**). Percentage change in tumor volume during treatment correlated with ^89^Zr-trastuzumab uptake (**d**). T, tumor; L, liver; d, days; %(ID)/g, injected dose per gram of tissue

Mice bearing palpable BT-474 tumors were dosed with dasatinib for 7 and 14 days and imaged with ^18^F-FDG 24 h prior to administration of ^89^Zr-trastuzumab (Fig. 2b). Tumor uptake of ^18^F-FDG was not statistically different between untreated mice (3.60 ± 1.51% ID/g) and treated cohorts at 7 days (3.86 ± 0.59 %ID/g, *p* = 0.99) and 14 days (4.63 ± 0.21 %ID/g, *p* = 0.80) (Fig. 3a). In comparison, ^89^Zr-trastuzumab exhibited a significant decrease in tumor accumulation in both treated groups (7 days, 11.05 ± 2.10 %ID/g, *p* < 0.0001; 14 days, 9.2 ± 1.85 %ID/g, *p* < 0.0001) compared to untreated tumors (17.88 ± 2.18 %ID/g) (Fig. 3b). No significant difference in probe uptake was observed between 7-day and 14-day treatments (*p* = 0.39) (Fig. 3c). A significant positive correlation was achieved (Fig. 3d, Additional file 7: Table S4) wherein a decrease in tumor volume, measured prior and after treatment, matched a reduction in PET tumor VOI (*r* = 0.85, *p* = 0.001).

**Fig. 4:**
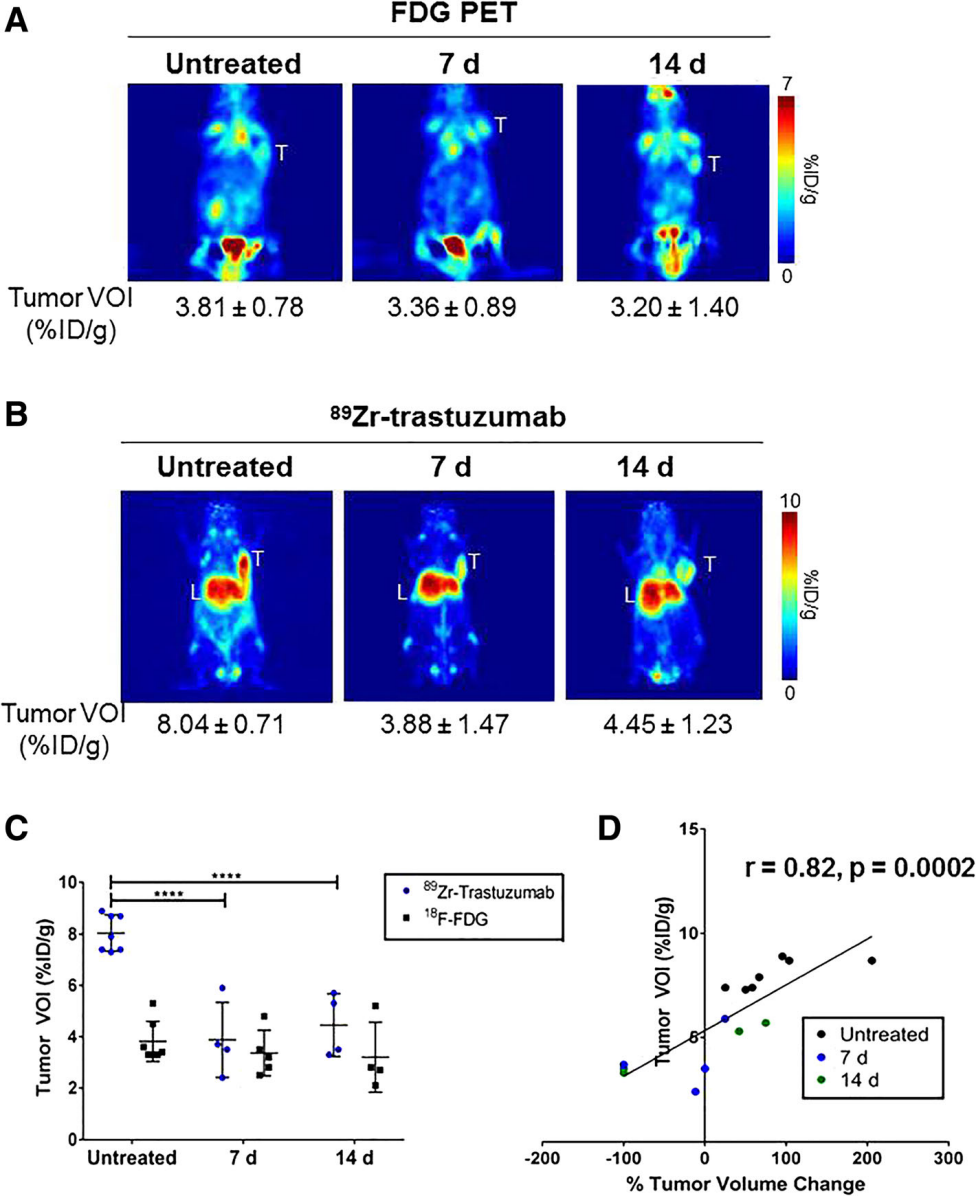
^89^Zr-trastuzumab positron emission tomography (PET) predicts tumor response to treatment in JIMT-1 xenografts. Untreated (left) and 7-day (middle) or 14-day (right) treated JIMT-1 tumors imaged with fluorodeoxyglucose (FDG) (**a**). The same group of mice were imaged with ^89^Zr-trastuzumab after 48 h post injection (**b**). Volumes of interest (VOIs) drawn on the tumors displayed lower accumulation of ^89^Zr-trastuzumab in treated groups compared to control but no change in FDG-PET tumor uptake was observed across the cohorts (**c**). Percentage change in tumor volume correlated with ^89^Zr-trastuzumab uptake (**d**). T, tumor; L, liver; d, days

In JIMT-1 tumor-bearing mice, FDG-PET did not distinguish between tumors in untreated groups (3.81 ± 0.78 %ID/g) and dasatinib-treated groups (7 days, 3.36 ± 0.89 %ID/g, *p* = 0.73; 14 days, 3.20 ± 1.37 %ID/g, *p* = 0.61) (Fig. 4a). In the same mice imaged with ^89^Zr-trastuzumab, tumor uptake displayed VOIs of 8.04 ± 0.71 %ID/g in the control, whereas there was a two-fold decrease in uptake in the treated groups at 7 days (3.88 ± 1.47 %ID/g, *p* < 0.0001) and 14 days (4.45 ± 1.23 %ID/g, *p* < 0.0001) (Fig. 4b). Tracer accumulation did not differ between 7-day and 14-day treatment (*p* = 0.71) (Fig. 4c). A strong association between ^89^Zr-trastuzumab PET uptake and tumor volume regression was recapitulated in this tumor model (*r* = 0.82, *p* = 0.0002) (Fig. 4d, Additional file 8: Table S5). Collectively, these results suggest that ^89^Zr-trastuzumab can effectively delineate tumors responsive to dasatinib treatment.

### Ex vivo analysis of JIMT-1 or BT-474 tumors

**Fig. 5:**
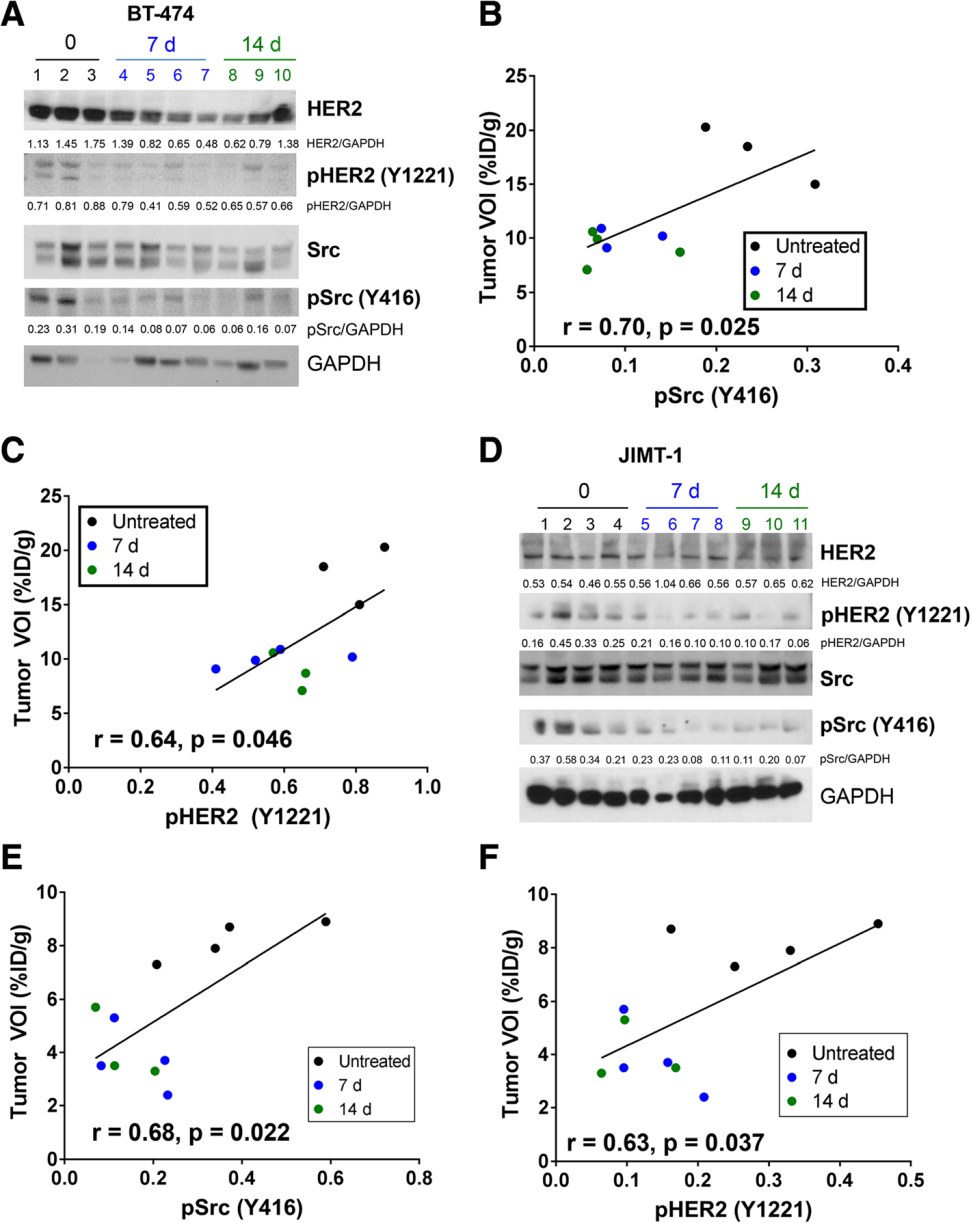
Ex vivo validation on excised BT-474 and JIMT-1 tumors confirm positron emission tomography (PET) uptake. Western blots were performed for human epidermal growth factor receptor 2 (HER2), Src, and phosphorylated Src (pSrc) (Y416) expression using BT-474 tumor lysates (**a**); a plot of the pSrc (Y416) densitometry showed a linear relationship with ^89^Zr-trastuzumab PET uptake (**b**). A plot of the pHER2 (Y1221) densitometry showed a linear relationship with ^89^Zr-trastuzumab PET uptake (**c**). Similarly, JIMT-1 tumors obtained after PET was lysed and analyzed for protein expression using western blots (**d**). pSrc (Y416) also demonstrated a direct linear correlation with ^89^Zr-trastuzumab PET uptake in JIMT-1 tumors (**e**). A plot of the pHER2 (Y1221) densitometry showed a linear relationship with ^89^Zr-trastuzumab PET uptake (**f**). All densitometry values were obtained as the ratio of total protein to glyceraldehyde-3-phosphate dehydrogenase (GAPDH)

After imaging, tumors were removed for ex vivo validation of the PET readout. From the immunoblot analysis, BT-474 tumors had moderately decreased levels of total Src upon treatment with dasatinib, whereas its activity was mitigated 2.6-fold as displayed by pSrc (Y416) levels in both 7-day and 14-day treated cohorts (Fig. 5a). Additionally, there was a decrease in total HER2 as assessed by densitometry after 7-day and 14-day treatments (Fig. 5a). A positive correlation was observed between tumor VOI values and pSrc (Y416) (*r* = 0.70, *p* = 0.025) (Fig. 5b) and pHER2 (*r* = 0.64, *p* = 0.046) (Fig. 5c) (measured by densitometry) for BT-474.

Treated and control JIMT-1 tumors did not show a difference in total HER2 or Src expression; however, a noticeable decrease in both pSrc and pHER2 after 7-day and 14-day treatments was displayed (Fig. 5d). Moreover, there was significant, positive association between pSrc (Y416) (*r* = 0.68, *p* = 0.022) and ^89^Zr-trastuzumab tumor VOI (Fig. 5e). A direct relationship between dephosphorylated HER2 and tracer uptake in the tumor was also demonstrated (*r* = 0.63, *p* = 0.037) (Fig. 5f).

**Fig. 6:**
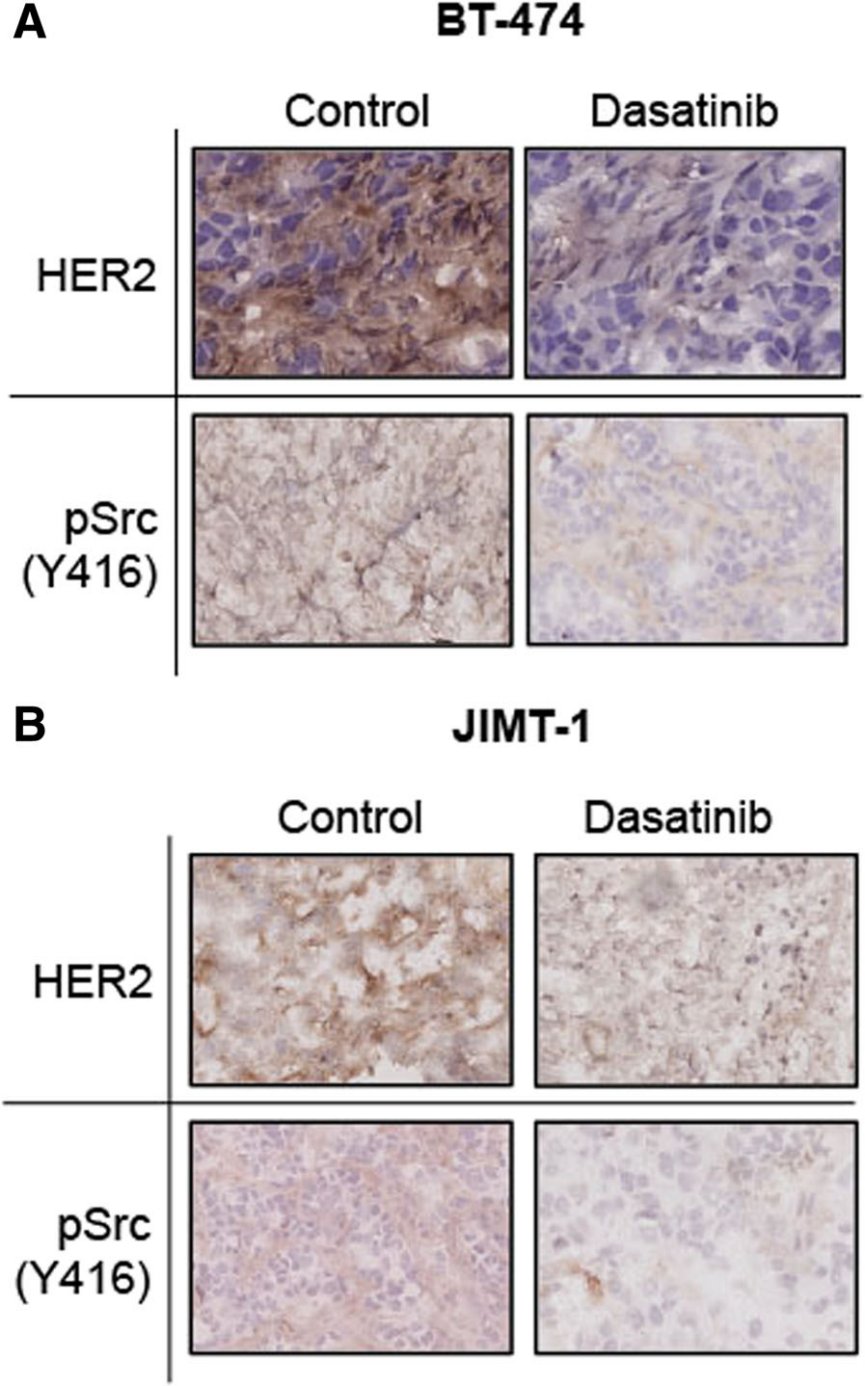
Immunohistochemical analysis (IHC) on excised BT-474 and JIMT-1 tumors show human epidermal growth factor receptor 2 (HER2) and pSrc (Y416) changes. IHC (× 40 magnification) was performed on excised BT-474 (**a**) and JIMT-1 (**b**) tumors showing HER2 (top) and phosphorylated Src (pSrc) (Y416, bottom) expression with (right) and without (left) dasatinib treatment (**a**)

IHC was performed to visualize subcellular localization of HER2 and pSrc (Y416) in excised tumors. Untreated BT-474 tumors showed strong, positive membranous HER2 staining (Fig. 6a, top left panel), whereas, predominant cytoplasmic HER2 localization was exhibited in tumors treated for 14 days. (Fig. 6a, top right). Less pSrc (Y416) staining was observed in treated tumors (Fig. 6a, bottom right) compared to control (Fig. 6a, bottom left). Control JIMT-1 tumors exhibited lower expression of membrane-localized HER2 (Fig. 6b, top left) compared to BT-474 but translocation to cytoplasmic regions was observed in treated sections (Fig. 6b, top right). Higher pSrc (Y416) staining waas displayed in control (Fig. 6b, bottom right) versus dasatinib-treated tumor sections (Fig. 6b, bottom left).

## Discussion

Trastuzumab has been the standard of care in HER2+ breast cancer for two decades [19]. Unfortunately, about half of patients with HER2-overxpressing breast cancer do not respond to trastuzumab due to de novo and acquired resistance mechanisms [20]. The non-receptor tyrosine kinase Src was shown to be one of the key modulators of trastuzumab response, and is an important downstream node of multiple trastuzumab resistance pathways [5–7, 18, 20]. Targeting Src with dasatinib in vitro re-sensitized trastuzumab-resistant cell lines, suggesting this pathway as a strategy to overcome resistance [6]. Using the Src inhibitor PP2, Fan et al. demonstrated that in vitro cellular Src modulation of trastuzumab-sensitive HER2+/estrogen receptor (ER)-negative SKBr3 cells decreased HER2 levels after 7 days and abrogated the expression completely after 14 days of treatment [9]. Further evidence identified improved antitumor effects in trastuzumab-resistant gastric xenografts compared to parental implants upon treatment with bosutinib, a Src-specific drug [21].

These preclinical findings were substantiated in patient studies implicating Src hyperactivity with trastuzumab resistance [6, 10]. In fact, tumors with high levels of phosphorylated Src at the Y416 residue presented with a lower clinical response rates, higher progressive disease, and shorter survival rates after trastuzumab treatment, compared to those with lower phosphorylated Src (Y416) levels [6, 10]. With this strong body of evidence, Src has become an appealing therapeutic target in the clinic. The GEICAM 2010/04 study (NCT01306942), in particular, initiated a safety and proof-of-concept synergistic study, which included dasatinib in combination with standard-of-care trastuzumab and paclitaxel [13]. Through pathology of sequential patient skin biopsies, reduction of pSrc was observed post-dasatinib treatment, with lower expression found with combined trastuzumab treatment [13]. The main concern with pSrc (Y416) as a biomarker of response lies in utilizing invasive sequential biopsies, which does not provide real-time information on the status of the tyrosine kinase.

Recently, Veach et al. reported the development and in vitro biological and in vivo pharmacologic activity of a ^18^F-labeled analog of dasatinib [22]. Dosimetric and pharmacokinetic profiles were investigated in preclinical studies [23] and are currently being investigated in clinical trials (NCT01916135). This method has its limitations since this can potentially miss functional effects upstream or downstream of the Src signaling pathway. The underlying relationship between Src and aberrant HER2 signaling provided us the impetus to examine ^89^Zr-trastuzumab PET as a surrogate predictive marker of dasatinib treatment.

Using ^89^Zr-trastuzumab as a surrogate marker of targeted inhibition of effector molecules downstream of the HER2 signaling pathway has been conceptually proven, for example, with Hsp90 inhibition [24]. Currently, this imaging probe is investigated in the clinic not only for diagnostic and staging purposes but also as a marker of response to other targeted treatments (NCT01081600 for AUY922 HSP90 inhibitor, NCT01565200 for T-DM1) [25]. To the best of our knowledge, this is the first study that demonstrated the potential of ^89^Zr-trastuzumab PET to monitor Src response to dasatinib treatment in trastuzumab-sensitive and trastuzumab-refractory breast cancer xenografts with proven Src activity. Specifically, we have shown that ^89^Zr-trastuzumab detects reduced functional HER2 through a concomitant decrease in internalization of the tracer after 6 h (BT-474) or 48 h (JIMT-1) of dasatinib treatment. The lower 89Zr-trastuzumab internalization was coupled with lower total HER2 present on the membrane, confirmed by ^89^Zr-trastuzumab binding experiments and western blots of pHER2(Y1221/1222). To date, limited information is known about the association between HER2 receptor internalization and dephosphorylation, which requires further study [26]. From our in vivo studies*,* a strong positive correlation was demonstrated between ^89^Zr-trastuzumab tumor uptake and tumor regression, changes in pSrc at the Y416 residue, and autophosphorylated HER2 at the Y1221/1222 residue. Importantly, the HER2-specific tracer detected these molecular events, where FDG, the gold standard PET imaging agents, has failed. Our histology studies encompassing decreased pSrc (Y416) with concomitant lower membranous HER2 further support and validate the ^89^Zr-trastuzumab PET readout. Taken together, ^89^Zr-trastuzumab can potentially be explored and utilized to assess dasatinib therapy in HER2+ breast cancer patients with elevated Src activity. However, it is worth noting that our studies are limited to single-agent Src inhibition; the utility of ^89^Zr-trastuzumab PET in combined therapies including dasatinib in HER2+ breast cancer still warrants further investigation.

## Conclusions

^89^Zr-trastuzumab can potentially delineate changes in Src activity and status in HER2+ breast cancer in both trastuzumab-sensitive and trastuzumab-resistant phenotypes.

## Additional files

Additional file 1: **Table S1.** Antibodies and dilutions used for each study. (JPG 425 kb)

Additional file 2: **Figure S1.** ^89^Zr-trastuzumab retains immunoreactivity in BT-474. Immunoreactivity of ^89^Zr-trastuzumab showed retained reactivity with *r*^2^ = 0.96. (JPG 173 kb)

Additional file 3: **Figure S2.** ^89^Zr-trastuzumab is specific for HER2 in vitro and in vivo. BT-474, JIMT-1 and MDA-MB-468 cells were incubated with 100 ng ^89^Zr-trastuzumab alone or co-incubated with 25-fold unlabeled trastuzumab before being lysed and radioactivity was measured using a gamma counter. (A) Nude mice bearing MDA-MB-468, BT-474 or JIMT-1 tumors were imaged with ^89^Zr-trastuzumab 48 h p.i. (B) Tumor VOIs showing significant uptake in HER2+ tumors, but no uptake in MDA-MB-468 (HER2-) tumors (C). (TIF 4980 kb)

Additional file 4: **Figure S3.** ^89^Zr-trastuzumab tumor uptake compared to isotype matched control. Mice bearing BT-474 and JIMT-1 tumors were injected with ^89^Zr-IgG or ^89^Zr-trastuzumab and tumors were removed 48 h p.i. and measured using a gamma counter. In both cell lines, specific ^89^Zr-trastuzumab uptake was significantly higher than isotype control IgG. (JPG 267 kb)

Additional file 5: **Table S2.** ^89^Zr-trastuzumab and ^89^Zr-IgG biodistribution in BT-474 tumors. (JPG 117 kb)

Additional file 6: **Table S3.** ^89^Zr-trastuzumab and ^89^Zr-IgG biodistribution in JIMT-1 tumors. (JPG 116 kb)

Additional file 7: **Table S4.** ^89^Zr-trastuzumab tumor VOI, pSrc (416) densitometry, and pHER2 (Y1221/1222) densitometry values for BT-474. (JPG 64 kb)

Additional file 8: **Table S5.** ^89^Zr-trastuzumab tumor VOI, pSrc (416) densitometry, and pHER2 (Y1221/1222) densitometry values for JIMT-1. (JPG 68 kb)

## Abbreviations

DFO: p-Benzyl-isothiocyanate-desferrioxamine
DMEM: Dulbecco’s modified Eagle’s medium
DMSO: Dimethyl sulfoxide
FBS: Fetal bovine serum
^18^F-FDG: ^18^Fluorine-Fluorodeoxyglucose
GAPDH: Glyceraldehyde-3-phosphate dehydrogenase
HER2: Human epidermal growth factor receptor 2
HRP: Horseradish peroxidase
IC_50_: Half maximal inhibitory concentration
IHC: Immunohistochemical analysis
iTLC: Instant thin layer chromatography
i.v.: Intravenously
kDa: KiloDalton
PBS: Phosphate-buffered saline
PET: Positron emission tomography
p.i.: Post-injection
pSrc: Phosphorylated Src
RTKs: Receptor tyrosine kinases
s.c.: Subcutaneously
TBST: Tris-buffered saline and Tween 20
VOI: Volume of interest
