# Supplementary figures and images for: Monitoring Src status after dasatinib treatment in HER2+ breast cancer with 89Zr-trastuzumab PET imaging
Source: Breast Cancer Res. 2018 Oct 25;20:130. doi: 10.1186/s13058-018-1055-2 (PMC6203283; doi:10.1186/s13058-018-1055-2)

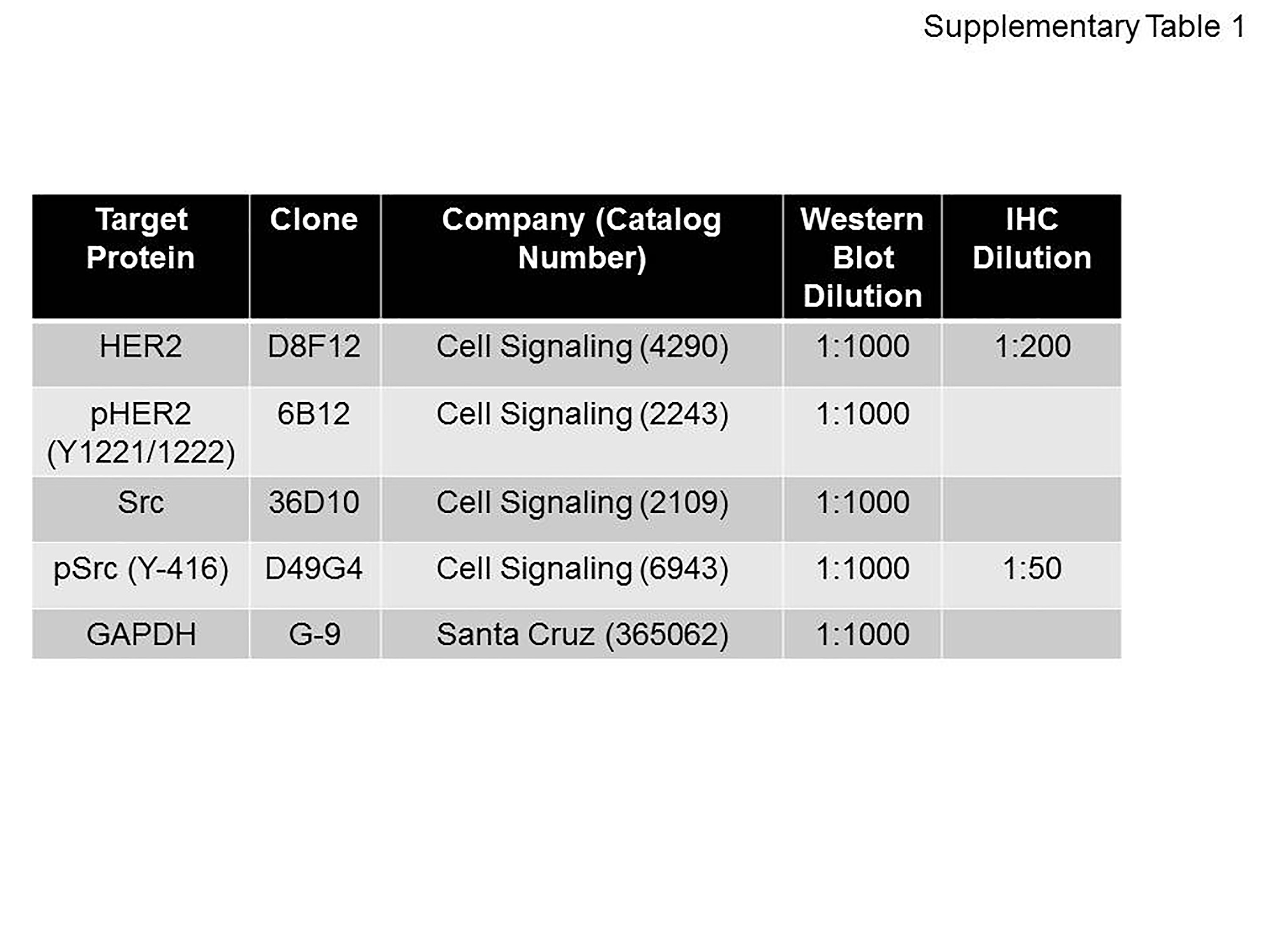

Supplement: Supplementary file 1 — Table S1. Antibodies and dilutions used for each study. (JPG 425 kb) [file 13058_2018_1055_MOESM1_ESM.jpg]

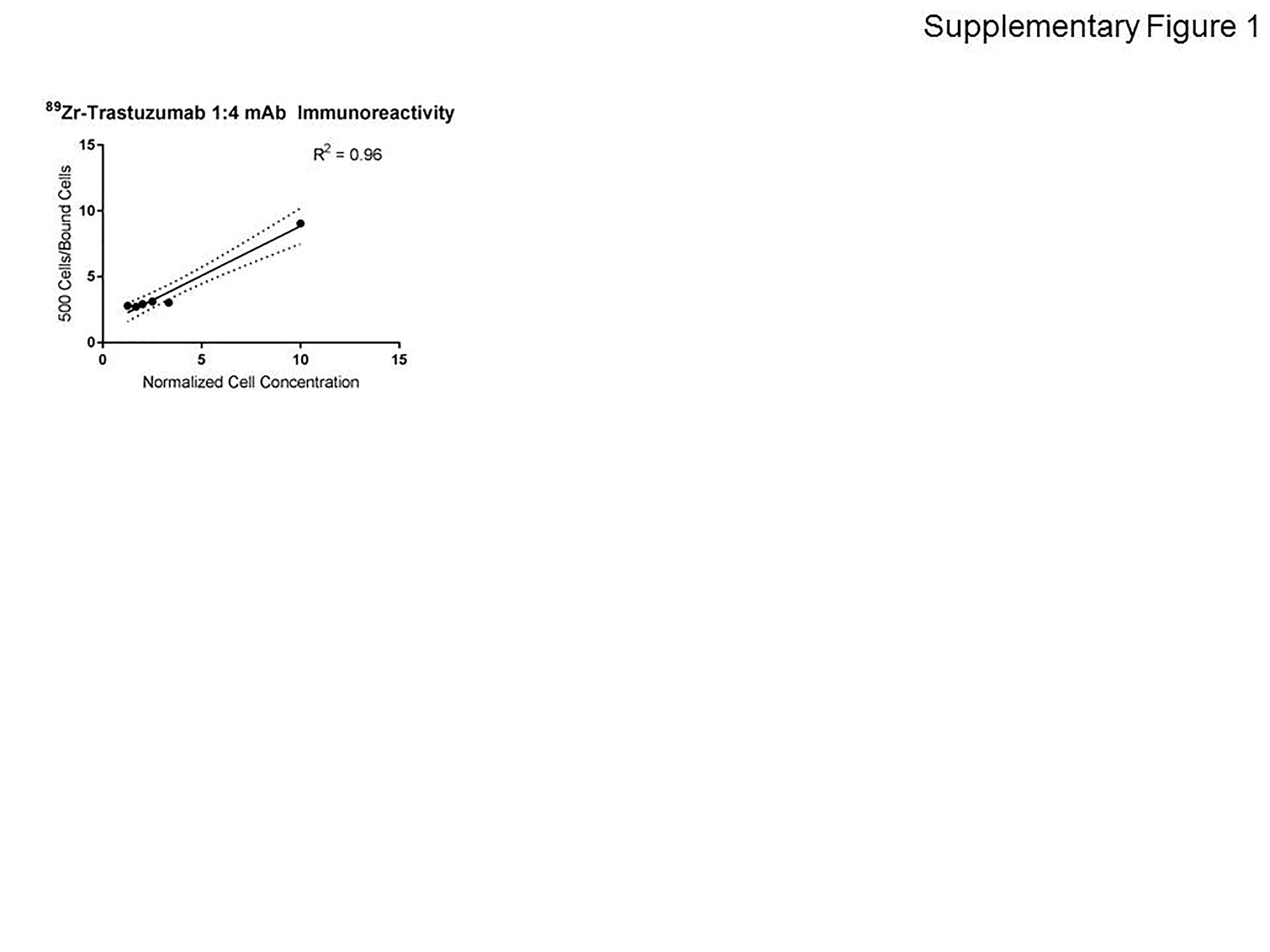

Supplement: Supplementary file 2 — Figure S1. 89Zr-trastuzumab retains immunoreactivity in BT-474. Immunoreactivity of 89Zr-trastuzumab showed retained reactivity with r2 = 0.96. (JPG 173 kb) [file 13058_2018_1055_MOESM2_ESM.jpg]

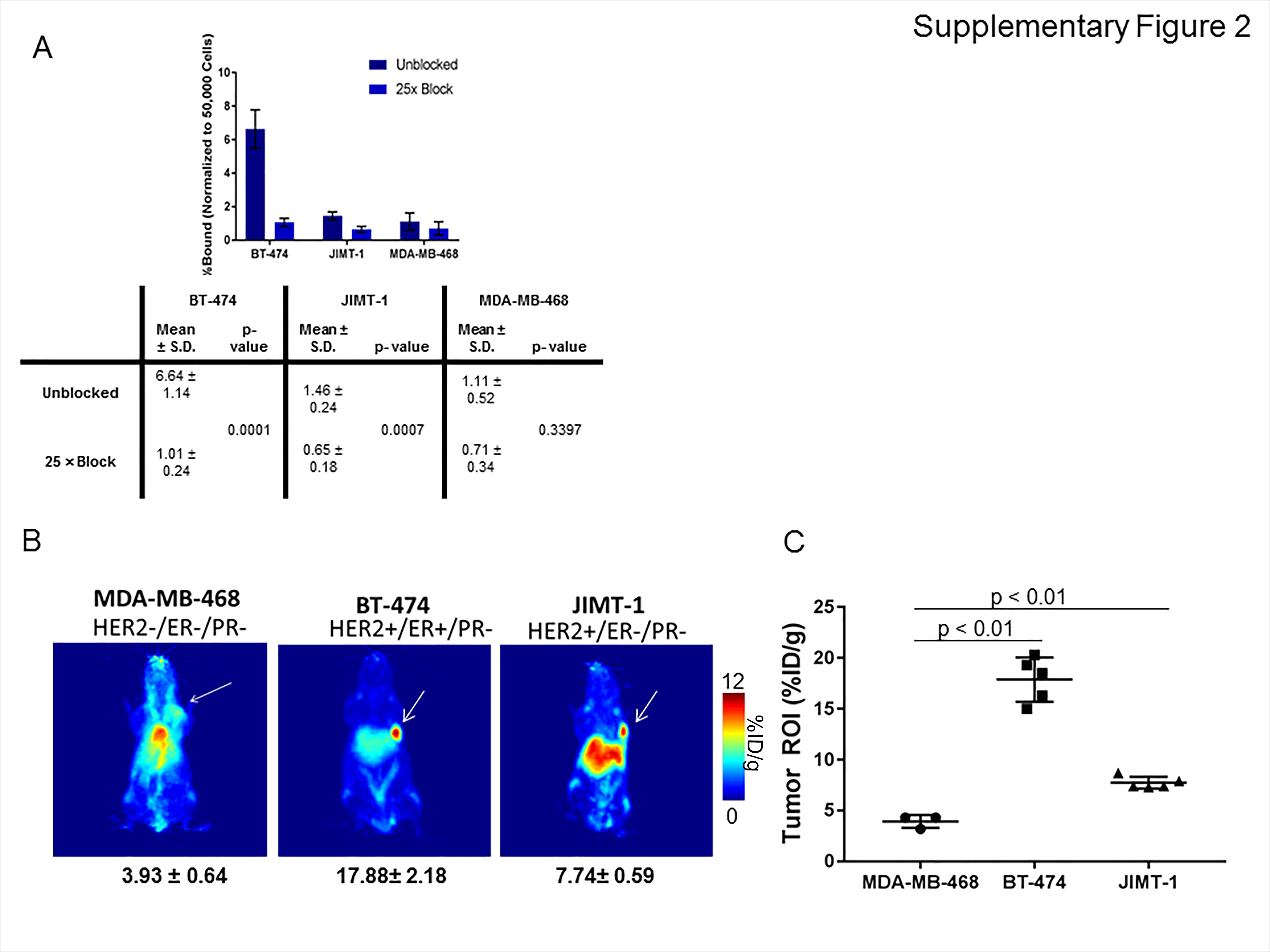

Supplement: Supplementary file 3 — Figure S2. 89Zr-trastuzumab is specific for HER2 in vitro and in vivo. BT-474, JIMT-1 and MDA-MB-468 cells were incubated with 100 ng 89Zr-trastuzumab alone or co-incubated with 25-fold unlabeled trastuzumab before being lysed and radioactivity was measured using a gamma counter. (A) Nude mice bearing MDA-MB-468, BT-474 or JIMT-1 tumors were imaged with 89Zr-trastuzumab 48 h p.i. (B) Tumor VOIs showing significant uptake in HER2+ tumors, but no uptake in MDA-MB-468 (HER2-) tumors (C). (TIF 4980 kb) [file 13058_2018_1055_MOESM3_ESM.tif]

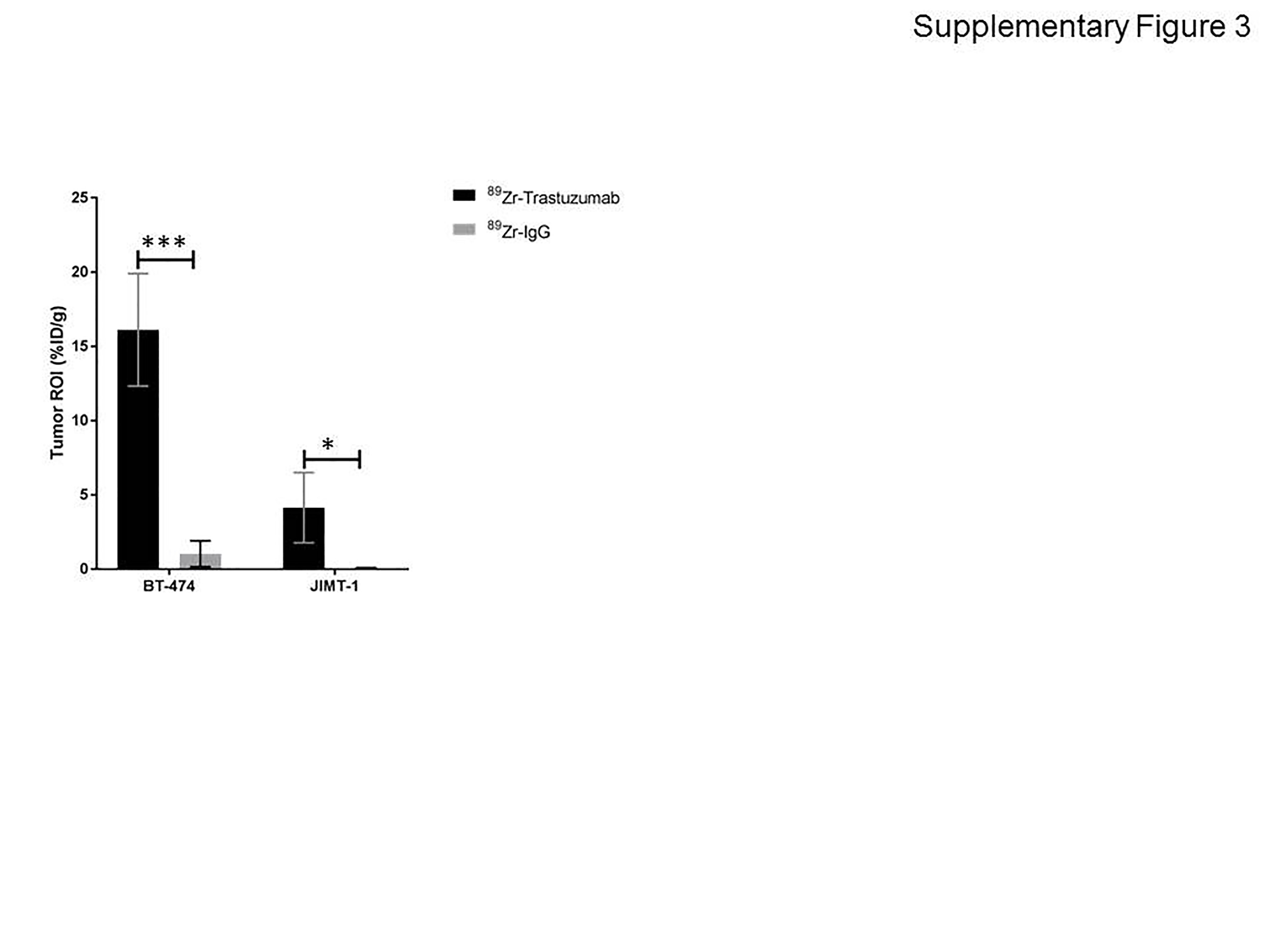

Supplement: Supplementary file 4 — Figure S3. 89Zr-trastuzumab tumor uptake compared to isotype matched control. Mice bearing BT-474 and JIMT-1 tumors were injected with 89Zr-IgG or 89Zr-trastuzumab and tumors were removed 48 h p.i. and measured using a gamma counter. In both cell lines, specific 89Zr-trastuzumab uptake was significantly higher than isotype control IgG. (JPG 267 kb) [file 13058_2018_1055_MOESM4_ESM.jpg]

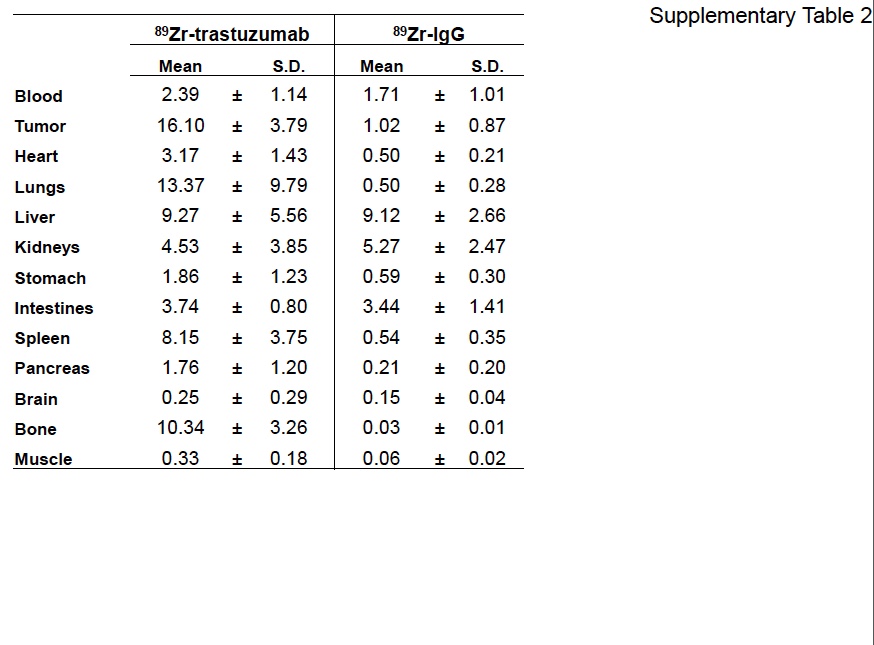

Supplement: Supplementary file 5 — Table S2. 89Zr-trastuzumab and 89Zr-IgG biodistribution in BT-474 tumors. (JPG 117 kb) [file 13058_2018_1055_MOESM5_ESM.jpg]

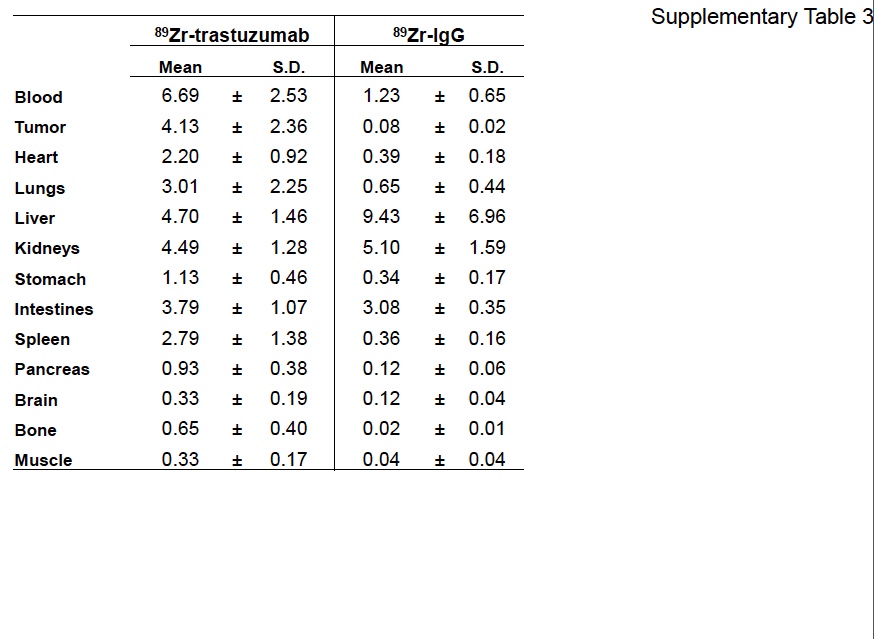

Supplement: Supplementary file 6 — Table S3. 89Zr-trastuzumab and 89Zr-IgG biodistribution in JIMT-1 tumors. (JPG 116 kb) [file 13058_2018_1055_MOESM6_ESM.jpg]

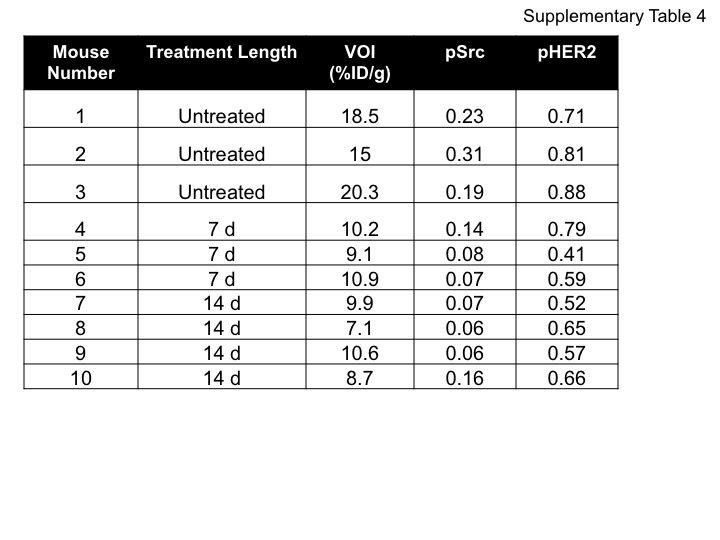

Supplement: Supplementary file 7 — Table S4. 89Zr-trastuzumab tumor VOI, pSrc (416) densitometry, and pHER2 (Y1221/1222) densitometry values for BT-474. (JPG 64 kb) [file 13058_2018_1055_MOESM7_ESM.jpg]

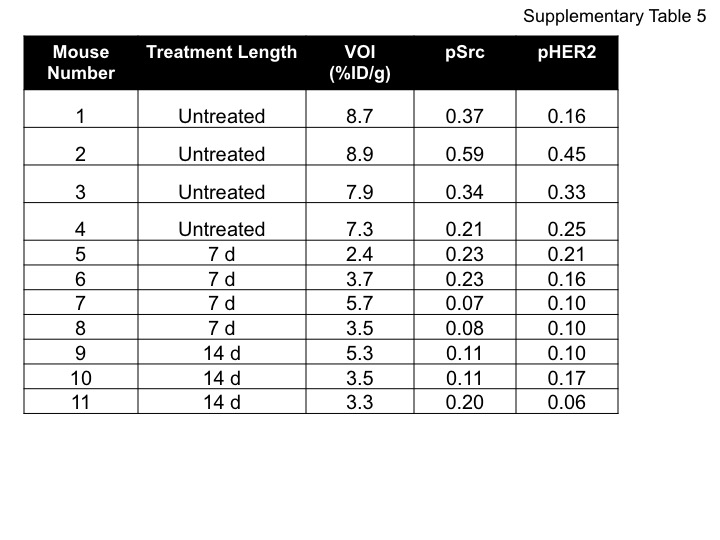

Supplement: Supplementary file 8 — Table S5. 89Zr-trastuzumab tumor VOI, pSrc (416) densitometry, and pHER2 (Y1221/1222) densitometry values for JIMT-1. (JPG 68 kb) [file 13058_2018_1055_MOESM8_ESM.jpg]
